# Supplementary material for: Impact of COVID-19-related knowledge on protective behaviors: The moderating role of primary sources of information
Source: PLoS One. 2021 Nov 29;16(11):e0260643. doi: 10.1371/journal.pone.0260643 (PMC8629273; doi:10.1371/journal.pone.0260643)
Supplement: S1 Table — (DOCX) [file pone.0260643.s001.docx]

**Table S1. Questionnaires used in the survey**

| Question | Response Type | Correct Response |
| --- | --- | --- |
| Knowledge | | |
| Coronavirus is a contagious disease. | 0=FALSE  1=TRUE | TRUE |
| A person infected with Coronavirus is not contagious until after symptoms appear. | 0=FALSE  1=TRUE | FALSE |
| Coronavirus cannot be spread through sneezing and coughing. | 0=FALSE  1=TRUE | FALSE |
| Currently, there is an FDA approved drug for treating individuals with Coronavirus. | 0=FALSE  1=TRUE | FALSE |
| Coronavirus can live on surfaces outside of the body for a few hours or several days. | 0=FALSE  1=TRUE | TRUE |
| There is no vaccine currently available to prevent infection with Coronavirus. | 0=FALSE  1=TRUE | TRUE |
| Children are at high risk for complications from Coronavirus. | 0=FALSE  1=TRUE | FALSE |
| Older people with other health conditions are more likely to die from Coronavirus. | 0=FALSE  1=TRUE | TRUE |
| People with Coronavirus can have no symptoms at all. | 0=FALSE  1=TRUE | TRUE |
| Most people with Coronavirus will have severe or critical symptoms. | 0=FALSE  1=TRUE | FALSE |
| Alcohol-based hand sanitizers cannot protect you from Coronavirus. | 0=FALSE  1=TRUE | FALSE |
| Coronavirus may be transmitted by mosquito bites. | 0=FALSE  1=TRUE | FALSE |
| Coronavirus originated from animals | 0=FALSE  1=TRUE | TRUE |
| Washing your hands frequently with soap and water. | 0=FALSE  1=TRUE | TRUE |
| Getting a flu shot. | 0=FALSE  1=TRUE | FALSE |
| Wearing a face mask. | 0=FALSE  1=TRUE | TRUE |
| Stop going to school/work. | 0=FALSE  1=TRUE | TRUE |
| Wiping potentially contaminated surfaces with a disinfectant. | 0=FALSE  1=TRUE | TRUE |
| Staying away from people who sneeze and cough. | 0=FALSE  1=TRUE | TRUE |
| Avoiding touching your eyes, nose and mouth. | 0=FALSE  1=TRUE | TRUE |
| Taking antibiotics. | 0=FALSE  1=TRUE | FALSE |
| Behaviors | | |
| Got a flu shot (or had my children get a flu shot) after hearing about Coronavirus. | 0=FALSE  1=TRUE | FALSE |
| Started using hand-sanitizer and/or washing my hands more often. | 0=FALSE  1=TRUE | TRUE |
| Started drinking more fluids and/or getting more rest. | 0=FALSE  1=TRUE | FALSE |
| Started taking antiviral and/or antibiotics. | 0=FALSE  1=TRUE | FALSE |
| Started taking dietary supplements (e.g., vitamins, probiotics). | 0=FALSE  1=TRUE | FALSE |
| Started cleaning and/or disinfecting things that I might touch (e.g., doorknobs, phone). | 0=FALSE  1=TRUE | TRUE |
| Started wearing rubber gloves in public. | 0=FALSE  1=TRUE | TRUE |
| Started taking more hot baths. | 0=FALSE  1=TRUE | TRUE |
| Started spending more time at home | 0=FALSE  1=TRUE | TRUE |
| Started wearing a face mask or cloth face covering when I leave home | 0=FALSE  1=TRUE | TRUE |
| Started practicing social distancing | 0=FALSE  1=TRUE | TRUE |
| Avoided seeking medical or dental care for other health concerns | 0=FALSE  1=TRUE | TRUE |
| Avoided leaving home except for food or medical supplies | 0=FALSE  1=TRUE | TRUE |
