# Supplementary material for: Impact of COVID-19-related knowledge on protective behaviors: The moderating role of primary sources of information
Source: PLoS One. 2021 Nov 29;16(11):e0260643. doi: 10.1371/journal.pone.0260643 (PMC8629273; doi:10.1371/journal.pone.0260643)
Supplement: S2 Table — (DOCX) [file pone.0260643.s002.docx]

**Table S2. Standardized residuals of chi-square test performed on the demographics of the study participants (n=6,518)**

|  | Total (n=6518) | Doctor or medical provider (n=1792) | Government or other official sources (e.g. CDC or WHO) (n=1948) | Traditional media (n=721) | New media (Social media, web surfing, podcasts, and etc,. (n=519) | Family, friends, and coworkers (n=309) | Religious leaders (n=3) | p-value |
| --- | --- | --- | --- | --- | --- | --- | --- | --- |
| Sex |  |  |  |  |  |  |  | <0.01 |
| Female | 3717 (57.6%) | -4.09 | 9.08 | 2.42 | -7.28 | -4.86 | 1.45 |  |
| Male | 2738 (42.4%) | 4.09 | -9.08 | -2.42 | 7.28 | 4.86 | -1.45 |  |
| Age group |  |  |  |  |  |  |  | <0.01 |
| 18-39 years old | 1078 (16.5%) | -4.09 | 5.23 | -1.28 | -0.44 | 0.01 | -0.77 |  |
| 40-59 years old | 2811 (43.1%) | -0.8 | 1.39 | -4.14 | 3.27 | 0.58 | 0.8 |  |
| 60+ years old | 2629 (40.3%) | 3.91 | -5.37 | 5.16 | -2.98 | -0.6 | -0.23 |  |
| Race |  |  |  |  |  |  |  | <0.01 |
| White, Non-Hispanic | 6012 (92.2%) | 1.06 | 0.7 | 1.38 | -2.42 | -2.34 | -1.78 |  |
| Non-White | 506 (7.8%) | -1.06 | -0.7 | -1.38 | 2.42 | 2.34 | 1.78 |  |
| Employment status |  |  |  |  |  |  |  | <0.01 |
| Employed | 2845 (56.2%) | -1.36 | 2.35 | -2.94 | 2.52 | -0.96 | -0.18 |  |
| Student/Unpaid work | 280 (5.5%) | -2.71 | 3.06 | -0.24 | -1.24 | 1.11 | -0.34 |  |
| Not working/Unemployed | 635 (12.5%) | -0.99 | 0.29 | 0 | 0.7 | 0.57 | -0.54 |  |
| Retired | 1300 (25.7%) | 3.71 | -4.49 | 3.46 | -2.74 | 0.07 | 0.79 |  |
| Educational attainment |  |  |  |  |  |  |  | 0.0602 |
| High school or less | 516 (13.9%) | -0.17 | 0.11 | -2.05 | 1.2 | 1.45 | -0.4 |  |
| Some college / Associate's degree | 1720 (46.5%) | 1.87 | -1.14 | -1.27 | -0.42 | 0.78 | 1.07 |  |
| Bachelor's degree or higher | 1463 (39.6%) | -1.79 | 1.08 | 2.75 | -0.42 | -1.82 | -0.81 |  |
| Political affiliation |  |  |  |  |  |  |  | <0.01 |
| Democrat | 1925 (38.3%) | -2.69 | 2.4 | 11.36 | -8.58 | -5.41 | -1.11 |  |
| Republican | 1222 (24.3%) | 0.13 | -2.01 | -5.53 | 5.06 | 5.51 | 2.5 |  |
| Other | 1072 (21.3%) | 1.3 | 0.31 | -4.84 | 3.56 | -0.56 | -0.74 |  |
| Prefer not to say | 809 (16.1%) | 1.96 | -1.18 | -3.17 | 1.47 | 1.35 | -0.62 |  |
