# Supplementary material for: Impact of COVID-19-related knowledge on protective behaviors: The moderating role of primary sources of information
Source: PLoS One. 2021 Nov 29;16(11):e0260643. doi: 10.1371/journal.pone.0260643 (PMC8629273; doi:10.1371/journal.pone.0260643)
Supplement: Attachment — Submitted filename: Review Report PONE-D-21-24232.docx [file pone.0260643.s004.docx]

**Review Report**

**PONE-D-21-24232**

Dear editor,

I am submitting the review report on Manuscript ID: PONE-D-21-24232 titled ‘**Impact of COVID-19-Related Knowledge on Protective Behaviors: The Moderating Role of Primary Sources of Information**’. The research study is related to the relationship between people’s COVID-19 information and protective behaviors and the influence of primary source of COVID-19 information on it. Different parametric and non-parametric statistical methods have been used for data analysis.

While the findings of the study are interesting and comprehensive, one major shortcoming of the statistical analysis is that the authors have used inferential methods on the basis of a non-probability sample (convenience sample). As inferences are often drawn / valid only if a probability sample is used, so the authors need to justify it. If authors have followed a previous work where inferences have been drawn on the basis of a non-probability sample, they must cite it. The authors must clear this point.

Moreover, the abstract has been divided into sub-sections. I suggest the authors to write a brief abstract as a single section. No need to divide the abstract into sub-sections.

Further, in the text, citations have been given using parenthesis. Use square brackets for citations.

**The authors must clear the above issues before I can recommend the paper for publication.**
