# Supplementary material for: Impact of COVID-19-related knowledge on protective behaviors: The moderating role of primary sources of information
Source: PLoS One. 2021 Nov 29;16(11):e0260643. doi: 10.1371/journal.pone.0260643 (PMC8629273; doi:10.1371/journal.pone.0260643)
Supplement: Attachment — Submitted filename: Revision2_PONE-D-21-24232_final.docx [file pone.0260643.s006.docx]

**Response to reviewers**

**PONE-D-21-24232**

Dear editor and reviewers,

On behalf of my co-authors, I would like to thank the reviewer(s) for their second round of feedback on our manuscript. Please find below an itemized list of our responses to the comments.

Hereby, we would like to also confirm that the material has not been and will not be submitted to any other journal for publication, as long as it is under consideration at PlosONE.

Please do not hesitate to reach out to me with questions.

Sincerely,

Yesim Tozan, PhD

Reviewer #1: The authors have successfully addressed most of my comments. I do have a few remaining questions.

1. The authors focus on primary information sources rather than number or type of sources. However, their introduction section does not mention primary information source (but rather, social media as a complementary source). I think the authors should explain why they chose to only focus on one source, what is the logical or theoretical justification for the decision.

🡪 Thank you for this comment. We expanded the text in Introduction to explain further and justify the focus on primary source of information along with references from the published literature. Please kindly refer to lines 68-72 and lines 82-84.

2. Thank you for conducting the Rasch analysis. While your results support the use of a Rasch scale over summary scores given the great fit statistics, I understand it might be too difficult to change the analyses at this stage.

🡪 Thank you for this feedback.

3. I understand the purpose of a Wilcoxon test, but the authors did not provide evidence that the data violate the assumptions of a t-test. They should add the sentence about the scores being left-skewed to the manuscript.

🡪 Thank you for this feedback. We added this explanation in lines 136-137 as follows: “*Pairwise Wilcoxon rank-sum test—a nonparametric alternative to the t test—was used, given the left-skewedness of the scores distribution, to compare the distribution of knowledge and behavior scores between each group.”*

4. The tables do not follow APA style.

🡪 Thank you for this feedback. We followed the SAMPL guideline (<https://www.equator-network.org/wp-content/uploads/2013/03/SAMPL-Guidelines-3-13-13.pdf>) for statistical reporting and PLOS ONE’s guideline (<https://journals.plos.org/plosone/s/tables>) for table formatting. We re-looked at both references to ensure correct reporting format and style and made following additional updates on both Table 1 and Table 2:

1) We added extra column, instead of using indents, to differentiate the variable name and subsequent category names.
2) We reported all the p-values equal to or greater than 0.001 as equalities and only reported values under 0.001 as inequalities.
3) Following your feedback, we formatted tables with the horizontal lines only to be compliant with APA style (<https://apastyle.apa.org/style-grammar-guidelines/tables-figures/tables>)
4) To improve legibility, we used bold text for the variable names and primary column titles.

Reviewer #3: COVID-19 is a global issue and these types of research studies are badly needed. I found this research study very interesting and worth publishable.

🡪 We thank you for your endorsement.
